# Retraction Note: Dissecting the role of novel EZH2 inhibitors in primary glioblastoma cell cultures: effects on proliferation, epithelialmesenchymal transition, migration, and on the pro-inflammatory phenotype

**DOI:** 10.1186/s13148-023-01595-6

**Published:** 2023-11-07

**Authors:** Giulia Stazi, Ludovica Taglieri, Alice Nicolai, Annalisa Romanelli, Rossella Fioravanti, Stefania Morrone, Manuela Sabatino, Rino Ragno, Samanta Taurone, Marcella Nebbioso, Raffaella Carletti, Marco Artico, Sergio Valente, Susanna Scarpa, Antonello Mai

**Affiliations:** 1 Department of Chemistry and Technologies of Drugs, Sapienza University of Rome, P.le A. Moro 5, 00185 Rome, Italy; 2 Department of Experimental Medicine, Sapienza University of Rome, P.le A. Moro 5, 00185 Rome, Italy; 3 Alchemical Dynamics s.r.l, 00125 Rome, Italy; 4 Department of Sense Organs, Sapienza University of Rome, P.le A. Moro 5, 00185 Rome, Italy; 5 Department of Radiologic, Oncological, and Anatomical and Pathological Sciences, Sapienza University of Rome, P.le A. Moro 5, 00185 Rome, Italy

**Retraction Note : Clinical Epigenetics (2019) 11:173** 10.1186/s13148-019-0763-5

The Editors-in-Chief have retracted this article. After publication, concerns were raised regarding image similarities in the western blot and invasion assay data presented in the figures. Specifically:

Fig. 5a H3K27me3 GL1 lanes 2 and 3 appear highly similar.

Fig. 5b H3K27me3 lane 1 appears highly similar to lane 5.

Fig. 7a and 9a appear to share the same beta-actin control blot.

Fig. 9b U-87 MC4040 and MC4041 images appear to originate from the same sample, although the stated treatments are different.

The authors have stated that the overlapping images in Fig. 9b were included by mistake. They have also provided partial raw data to address the western blot concerns; however, further checks by the Publisher have found that a number of western blot images presented in the article don't accurately represent the original data. The Editors-in-Chief therefore no longer have confidence in the presented data.

Susanna Scarpa does not agree to this retraction. Giulia Stazi, Rossella Fioravanti, Manuela Sabatino, Rino Ragno, Samanta Taurone, Marcella Nebbioso, Raffaella Carletti, Marco Artico, Sergio Valente and Antonello Mai have not responded to any correspondence from the publisher about this retraction. The publisher has not been able to obtain current email addresses for Ludovica Taglieri, Alice Nicolai, Annalisa Romanelli and Stefania Morrone.

